# Sclerostin, Osteocytes, and Wnt Signaling in Pediatric Renal Osteodystrophy

**DOI:** 10.3390/nu15194127

**Published:** 2023-09-25

**Authors:** Marciana Laster, Renata C. Pereira, Kathleen Noche, Barbara Gales, Isidro B. Salusky, Lauren V. Albrecht

**Affiliations:** 1 Department of Pediatrics, David Geffen School of Medicine, University of California, Los Angeles, CA 90024, USA; mlaster@mednet.ucla.edu (M.L.); rpereira@mednet.ucla.edu (R.C.P.); knoche@mednet.ucla.edu (K.N.); bgales@mednet.ucla.edu (B.G.); 2 Department of Pharmaceutical Sciences, School of Pharmacy, University of California, Irvine, CA 92697, USA; 3 Department of Developmental and Cell Biology, School of Biological Sciences, University of California, Irvine, CA 92697, USA

**Keywords:** Wnt signaling, CKD-MBD, children, sclerostin, bone biopsy, immunohistochemistry, immunofluorescence

## Abstract

The pathophysiology of chronic kidney disease-mineral and bone disorder (CKD-MBD) is not well understood. Specific factors secreted by osteocytes are elevated in the serum of adults and pediatric patients with CKD-MBD, including FGF-23 and sclerostin, a known inhibitor of the Wnt signaling pathway. The molecular mechanisms that promote bone disease during the progression of CKD are incompletely understood. In this study, we performed a cross-sectional analysis of 87 pediatric patients with pre-dialysis CKD and post-dialysis (CKD 5D). We assessed the associations between serum and bone sclerostin levels and biomarkers of bone turnover and bone histomorphometry. We report that serum sclerostin levels were elevated in both early and late CKD. Higher circulating and bone sclerostin levels were associated with histomorphometric parameters of bone turnover and mineralization. Immunofluorescence analyses of bone biopsies evaluated osteocyte staining of antibodies towards the canonical Wnt target, β-catenin, in the phosphorylated (inhibited) or unphosphorylated (active) forms. Bone sclerostin was found to be colocalized with phosphorylated β-catenin, which suggests that Wnt signaling was inhibited. In patients with low serum sclerostin levels, increased unphosphorylated “active” β-catenin staining was observed in osteocytes. These data provide new mechanistic insight into the pathogenesis of CKD-MBD and suggest that sclerostin may offer a potential biomarker or therapeutic target in pediatric renal osteodystrophy.

## 1. Introduction

Renal osteodystrophy (ROD) is a complex disorder of bone and mineral metabolism that affects virtually all pediatric patients with CKD. ROD is associated with adverse clinical outcomes that include bone loss, skeletal deformities, abnormal bone mineralization, and an increased risk of fractures and cardiovascular events [1]. These adverse effects have long-lasting consequences that persist into adulthood and contribute to the markedly reduced life expectancy of children with CKD. Given the limitations of biomarkers for bone remodeling in CKD [2,3], bone biopsy remains the gold standard for diagnosing abnormal bone turnover, mineralization, and volume. Current therapies rely on active vitamin D sterols that impact bone indirectly by targeting the levels of parathyroid hormone (PTH). However, the limitations of such interventions are underscored by the rising frequency of appendicular skeletal fractures in adult CKD patients, which has more than doubled from 1992 to 2009 [4,5]. Similar findings have also been shown in young adults with childhood-onset CKD, which further highlights the need to improve treatments by directly targeting the molecular basis of bone disease [6]. The earliest bone defects in CKD occur in osteocyte cells that are embedded within the bone matrix and secrete key factors to regulate bone remodeling [7,8]. Fibroblast growth factor 23 (FGF-23), dentin matrix protein 1 (DMP1), and sclerostin are osteocyte-secreted proteins that become elevated during the early stages of CKD when the circulating concentrations of calcium, phosphate (P), PTH, and active vitamin D are still within the normal range [7,8]. Given this, circulating osteocytic protein levels may offer a non-invasive, diagnostic biomarker of bone disease in CKD [9]. Elucidating the interactions between bone signaling and kidney function that shed light on the mechanistic underpinnings of disease will be required to facilitate the development of targeted therapeutic strategies.

Sclerostin is an osteocytic secreted factor that has been implicated in the progression of bone and kidney disease [10,11,12,13,14]. Sclerostin is a 22-kDa glycoprotein that is produced by osteocytes and regulates osteoblastic bone formation through paracrine mechanisms. Upon secretion, extracellular sclerostin binds to the Wnt receptor, low-density lipoprotein receptor-related protein (LRP5/6), and occludes the Wnt ligand binding sites that are required to initiate Wnt signaling and β-catenin transcriptional activity [15,16,17,18]. Among the many secreted Wnt inhibitors, sclerostin is a favorable candidate for drug design as it is primarily expressed in bone [19]. In fact, loss-of-function mutations in the sclerostin encoding gene, SOST, result in sclerosteosis and van Buchem disease (VBD), two autosomal recessive disorders that are characterized by hyperactive osteoblasts, high bone mass, and a thickening of the cortical bone [20,21]. Over forty years later, a new class of anabolic therapies has emerged that exploits sclerostin. Romosozumab is an antibody-based therapy that targets serum sclerostin to increase bone formation, and osteoporotic-treated patients had a lower incidence of fracture [22,23]. While promising, the indications for the use of anti-sclerostin therapies across the spectrum of bone disease in CKD remain unclear. Intriguingly, sclerostin has been implicated in adults with CKD-MBD [24,25]. Graciolli et al. provide evidence to support a link between bone remodeling, sclerostin expression, and Wnt signaling [25]. These data provide a strong premise to further interrogate the roles of sclerostin in CKD-MBD and to evaluate whether sclerostin levels could offer a biomarker or therapeutic target in adult and pediatric patients.

The present study was designed to characterize the relationship between bone and circulating sclerostin across a spectrum of pediatric CKD patients that includes pre-dialysis and dialysis (CKD 5D)-treated cohorts. These investigations combine the power of bone histomorphometry and biomarkers of CKD-MBD with immunofluorescence microscopy labeling approaches in un-decalcified bone biopsy specimens in situ.

## 2. Materials and Methods

### 2.1. Study Subjects

The population of this study consisted of 87 pediatric patients with CKD stages 2-5D, and serum was collected at the time of bone biopsy at UCLA between 2006 and 2015. This study was approved by the UCLA Institutional Review Board, and patients consented/assented under prior research protocols. Patients who had undergone kidney transplants, as well as individuals who were immunosuppressed or receiving growth hormone therapy, were excluded from these analyses. The inclusion criteria selected pediatric patients who were less than 21 years old, consented to allow stored serum for biomarker analysis, and had biopsies performed between 2006 and 2015. Additionally, all patients also had to have completed double tetracycline labeling.

### 2.2. Bone Biopsy, Serum Biochemistry, and Histomorphometry Measurements

Patients in this study underwent iliac crest bone biopsies, as previously described [26], and bone histomorphometric parameters were assessed in trabecular bone under 20× magnification using the OsteoMetrics system. Derived bone indices were calculated using standard formulas [27]. Bone pathology classification was performed according to the turnover, mineralization, and volume (TMV) system [28]. Histomorphometric reference values were established using double-tetracycline-labeled iliac crest specimens from 31 pediatric patients (ages 2.5–17 years) with normal kidney function obtained during elective orthopedic surgery [29]. Renal osteodystrophy diagnoses were assigned as follows: mild disease, mixed uremic osteodystrophy, osteitis fibrosa cystica, adynamic bone disease, or osteomalacia, as previously described [30,31].

Biochemical parameters of subjects included calcium (mg/dL), phosphorus (mg/dL), 25(OH) vitamin D (ng/mL), alkaline phosphatase (IU/L), intact parathyroid hormone (PTH) (pg/mL), sclerostin (pmol/L), intact FGF-23 (pg/mL), and C-terminal FGF-23 (RU/mL). Serum calcium (Ca), phosphorus (P), creatinine, and albumin were measured using an Olympus AU5400 analyzer (Woonsocket, RI, USA). PTH concentrations in EDTA plasma were measured by the first-generation immunometric (normal range: 10–65 pg/mL). FGF-23 levels were determined in EDTA plasma using a C-terminal assay and an intact assay (Quidel/Immunotopics, San Clemente, CA, USA). Serum sclerostin was measured from serum stored at −80 °C using human bioactive sclerostin (ELISA BI-20472 from Biomedica, Vienna, Austria) in CKD and healthy control (20–30 pmol/L) subjects.

### 2.3. Bone Immunohistochemistry (IHC) and Quantification of Sclerostin and FGF-23

IHC staining and quantification of sclerostin and FGF-23 were performed across the entire cohort of 87 patients. Iliac crest bone biopsy specimens from five adolescent and young adult subjects with normal renal function comprised the “healthy control” population. Bone IHC quantification was correlated with bone histomorphometric and biochemical parameters. Immunostaining of bone proteins was adapted from previous reports [8]. Briefly, bone tissue sections were de-plastified in xylene and chloroform, rehydrated in graded alcohol solutions, and partially decalcified in 1% acetic acid. Endogenous peroxidase activity was quenched in a 3% hydrogen peroxide/methanol solution. Non-specific binding was blocked in an avidin-biotin solution and in 5% normal horse serum with 1% bovine serum albumin. Sections were incubated with affinity-purified polyclonal goat anti-human FGF-23 or monoclonal anti-human sclerostin (1:500) overnight at 4 °C in a humidified chamber. Sections were then incubated with biotinylated antibodies towards goat, mouse, or rabbit; incubated for 30 min with a StreptABC Complex/HRP (Sigma-Aldrich, Burlington, MA, USA) kit that was followed by AEC subtract Chromogen; and counterstained with Mayer hematoxilin. Negative controls omitted primary antibodies. IHC was repeated on all specimens for staining reproducibility. FGF-23 was assessed in trabecular and sclerostin in both cortical and trabecular bone, and values were expressed as total sclerostin. Quantification of immunoreactivity was performed using the Ariol scanning system, and all slides were scanned at 20× magnification with a red filter and digitized. Analyzed fields, expressed as pixels/mm^2^, were manually selected to avoid areas with tissue damage [8].

### 2.4. Analysis of Wnt Signaling Activity

Immunofluorescence (IF) analyses were performed to assess Wnt activity, and comparisons were made across a subset of bone biopsies from 12 patients, selected based on serum sclerostin levels, and 3 healthy controls; six patients had CKD stages 2–4, and six were treated with dialysis. Within each group (CKD 2–4 and dialysis), three had serum sclerostin greater than 50 pmol/L and three below 37 pmol/L (normal range 10–30 pmol/L). Primary antibodies used included sclerostin and β-catenin, in phosphorylated (phos) or unphosphorylated (active) forms (Cell Signaling, Danvers, MA, USA; D13A1), and secondary antibodies included an AlexaFluor 568 or AlexaFluor 488. DAPI stained the nucleus [32]. Images were acquired on a Zeiss Imager Z.1 microscope with Apotome (Gottingen Germany) and Image J (FIJI) was used to normalize brightness and contrast across primary antibody channels. Quantification of sclerostin, phos-β-catenin, or active β-catenin-positive osteocytes is shown as the percentage of the total cell number marked by DAPI using matching bone area regions. For each condition, entire bone section areas were quantified per area for osteocyte protein expression analyses. Each image depicts osteocytes that were representative of the whole population per bone tissue section (n = 230–300 cells per condition) with scale bars of 50 μm. Image magnification was performed at 20× and bone unit areas were used for normalization. Background fluorescence was consistently subtracted. In order to rule out any differences, bone IF quantification was performed using biopsies that were processed together. Pearson’s assessed sclerostin and β-catenin colocalization relative to total sclerostin-positive cells. Figures show representative images and normalized fluorescence measurements and are presented as the mean ± SEM.

### 2.5. Statistical Analysis

Patient characteristics at the time of bone biopsy are reported as median (interquartile range) and frequency (percentage). The Mann-Whitney U test was used to assess between-group differences in continuous variables. The Chi-Square test and Fischer exact, where appropriate, were used to assess the difference in categorical variables between the two groups. Spearman rank correlation coefficients were used to assess the relationships between sclerostin and traditional biochemical variables, or histomorphometry parameters. For the reason that serum phosphorus concentrations vary with age in healthy children [33], we expressed the phosphorus value for each participant as a z score relative to age-matched values in 493 healthy children 1–20 years old. The primary variables of interest were serum sclerostin and quantitative bone sclerostin. Given the interplay between sclerostin and PTH, sclerostin-to-PTH ratios are shown for associations with biochemical and bone parameters. Simple logistic regression analysis was used to assess associations between the primary markers of interest and categorical outcomes of the TMV system (high turnover vs. non-high turnover, low turnover vs. non-low turnover, and abnormal mineralization vs. normal mineralization). Categorical outcomes were defined as follows: high turnover includes bone formation rate (BFR)/bone surface (BS) greater than 75 μm^3^/μm^2^/year or evidence of fibrosis; low turnover includes BFR/BS less than 10 μm^3^/μm^2^/year with decreased osteoblast and osteoclast cell activity; abnormal mineralization includes the combination of osteoid accumulation (elevated osteoid thickness and volume) and mineralization timing (elevated mineralization lag time and/or osteoid maturation time) [34]. All statistical analyses were performed using SAS version 9.4, and all tests were two-sided. A probability of type I error < 5% was considered statistically significant, and ordinary *p* values were reported.

## 3. Results

### 3.1. Cohort Characteristics

Patient demographics are presented in Table 1. The median (IQR) age of the cohort was 17 (14, 20), and 69% of the cohort were male. The majority of the cohort (66%) were of self-reported (or parent-reported) Hispanic ethnicity. 39% of the cohort had pre-dialysis. CKD and CKD stages 2 and 3 were the most prevalent CKD stages (82%). The remainder of the population (61%) had CKD stage 5 treated with dialysis (CKD 5D); 55% were treated with peritoneal dialysis and 45% with hemodialysis. The most common cause of CKD in this cohort was congenital anomalies of the kidney and urinary tract (CAKUT, 44%) or glomerulonephritis (35%).

### 3.2. Biochemical Characteristics

Biochemical parameters of pre-dialysis CKD and CKD 5D patients are displayed in Table 2. Levels of serum calcium were within the normal range in all subjects, while the median (IQR) phosphate z-score was greater than two standard deviations above the mean for age in CKD 5D (5.0 [2.9, 7.7]). Median (IQR) intact PTH was 411 (211, 991) pg/mL in CKD 5D, while only 95 (50, 159) pg/mL in pre-dialysis CKD participants. Similarly, plasma C-terminal (total) and intact FGF-23 levels were higher in CKD 5D than in pre-dialysis CKD. Across the entire CKD cohort, the median of sclerostin was 52.6 pmol/L, which was above that of our healthy controls (median (IQR) of 36.7 (28.4, 48.3) pmol/L with a range of 12.4–93.2 pmol/L). In the CKD 5D cohort, median serum sclerostin levels were also higher compared with pre-dialysis CKD patients.

### 3.3. Bone Histomorphometry in Pre-Dialysis CKD and CKD 5D

**Figure 1:**
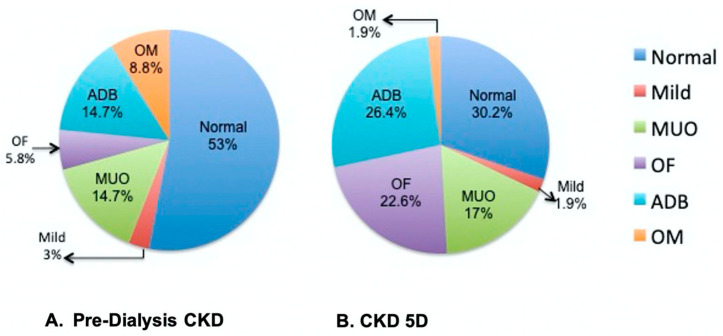
Distribution of Pediatric Renal Osteodystrophy Lesions in Pre-Dialysis CKD and CKD 5D Patients. Normal bone (Normal), M osteomalacia (OM), Mild bone lesion of secondary hyperparathyroidism (Mild), Mixed Uremic Osteodystrophy (MUO), osteitis fibrosa (OF), Adynamic Bone (ADB) and Osteomalacia (OM).

Bone histological parameters are summarized in Table 3. Within the entire cohort, 23% demonstrated high bone turnover disease, and 25% demonstrated low bone turnover. High bone turnover was more frequent in CKD 5D than in pre-dialysis CKD. Defective mineralization was present in 25% of the entire cohort, and the majority of the whole cohort demonstrated normal bone volume (63%). When assigned a ROD classification, 39% of the cohort had normal bone, 22% displayed adynamic bone (ADB), 16% had osteitis fibrosa, 16% had mixed uremic osteodystrophy (MUO), 4.7% had osteomalacia (OM), and 2.3% had a mild lesion of secondary hyperparathyroidism. In the pre-dialysis CKD cohort, the most frequent ROD subtypes were ADB (14.7%) and MUO (14.7%) (Figure 1A). The CKD 5D cohort had larger fractions of ADB (26.4%), OF (22.6%), and MUO (17%) (Figure 1B).

### 3.4. Correlations of Serum and Bone Sclerostin with Indices of Bone Histomorphometry

Using Spearman correlation coefficients, the relationship between circulating and bone sclerostin and categories of bone turnover and mineralization were analyzed by CKD severity. These analyses also included quantification of IHC staining of bone sclerostin and bone FGF-23.

### 3.5. Pre-Dialysis CKD Cohort

Serum sclerostin correlated inversely with the eroded surface and mineralization surface (Supplementary Table S1). Conversely, PTH correlated directly with the eroded surface, mineralization surface, and bone formation rate, as expected. PTH demonstrated an especially strong correlation with parameters of osteoid accumulation, including osteoid volume, thickness, and surface. PTH was correlated directly with both osteoid maturation and mineralization lag time, which supports a negative impact on mineralization. Bone sclerostin quantification was correlated with mineralization surface (*p* = 0.05). Additionally, a significant inverse association was found between serum sclerostin and intact PTH (Supplementary Table S1). Given this, associations were next evaluated between bone parameters and the ratio of serum sclerostin to PTH. While sclerostin alone correlated with few bone parameters, the sclerostin-to-PTH ratio values correlated moderately with multiple parameters (Supplementary Table S1). A higher sclerostin-to-PTH ratio (higher sclerostin for a given PTH) was negatively correlated with bone formation rate, eroded surface, osteoid accumulation, mineralization time, and mineralizing surface. This further fits the inhibitory role of sclerostin on bone formation. In logistic regression analysis, a higher sclerostin-to-PTH ratio predicted a lower probability for a mineralization defect [OR 0.86 (0.75, 0.98), *p* = 0.03]. While bone FGF-23 did not correlate with bone parameters, circulating intact FGF-23 directly correlated with bone formation rate and mineralizing surface.

### 3.6. CKD 5D Cohort

Serum sclerostin levels were inversely correlated with osteoid thickness, osteoid volume, and osteoid surface (Table 4). Notably, bone sclerostin is also inversely correlated with bone formation rate, osteoid thickness, and mineralization surface (Table 4). Similar to the pre-dialysis CKD cohort, PTH directly correlated with bone formation, eroded surface, osteoid volume, thickness, surface, and mineralizing surface, while the ratio of sclerostin to PTH inversely correlated with these parameters. In logistic regression, increased levels of bone sclerostin were associated with a decreased probability of high turnover disease [OR (CI) 0.71 (0.51, 0.99), *p* = 0.04], consistent with an inhibitory effect on bone formation. Furthermore, an increasing sclerostin/PTH ratio (higher sclerostin for a given PTH) was also associated with a decreased probability of high turnover disease [OR 0.72 (0.53, 0.98), *p* = 0.04]. Serum sclerostin inversely correlated with PTH and alkaline phosphatase levels, and only bone sclerostin correlated with bone FGF-23 (Table 4). Unlike the pre-dialysis CKD cohort, relationships between bone parameters and FGF-23, both bone and circulating, were apparent. For instance, bone FGF-23 was inversely correlated with bone formation, osteoid accumulation, mineralizing surface, and osteoid maturation time. Similarly, both intact and C-terminal FGF-23 were inversely correlated with osteoid volume, surface, and osteoid maturation time.

### 3.7. CKD Patients with Low Bone Turnover

Given the known inhibitory effect of sclerostin on bone formation, a sub-analysis of twenty-two patients with low bone formation was performed. Such patients had higher serum sclerostin levels than patients with non-low formation [median (IQR) 61.7 (41.6, 102.9) vs. 51 (36, 69), *p* = 0.04]; however, there was no difference in bone sclerostin between the low turnover group and non-low turnover participants. Moreover, in the low turnover group, serum sclerostin directly correlated with bone sclerostin (r = 0.44, *p* = 0.04), a relationship not evident in the population as a whole.

### 3.8. Serum Sclerostin Correlates with Bone Sclerostin and Wnt Signaling in CKD

**Figure 2:**
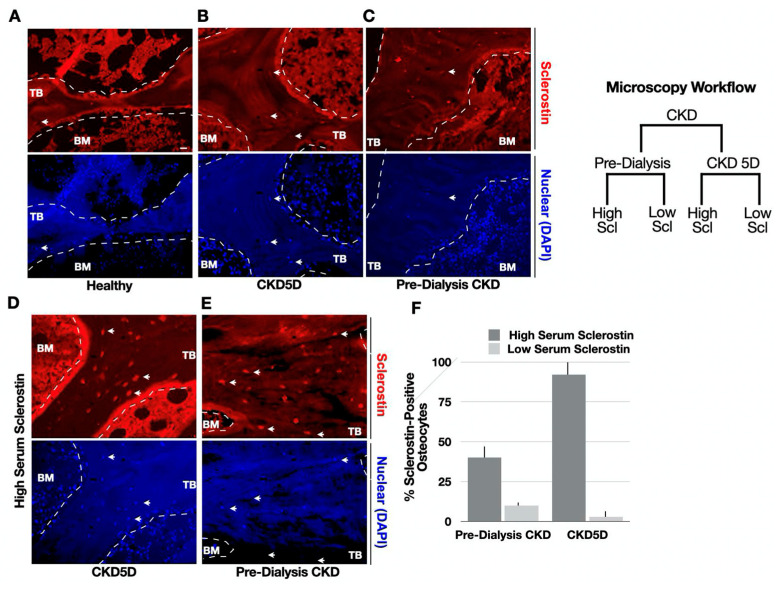
Representative Images of Sclerostin Immunofluorescence Analyses in CKD Bone. (**A**–**C**) Staining with an anti-sclerostin antibody (red) was assessed in both early-stage CKD (n = 6) and in dialysis CKD 5D patients (n = 6). As illustrated in the microscopy workflow diagram, patients were further designated into two groups with either high serum sclerostin (58–125 pmol/L) or low levels similar to healthy controls (18.6–36.0 pmol/L). DAPI was used as a nuclear marker (blue). (**D**,**E**) Bone stained from patients with high serum sclerostin. (**F**) Quantification of sclerostin-positive osteocytes normalized by total number of cells using bone unit area to normalize (n > 230 cells per condition). Dark grey bars indicate high serum sclerostin while light grey bars indicate low serum sclerostin. Trabecular (TB) and bone marrow (BM) are indicated. Arrows denote corresponding cells in red and blue channels.

We next set out to evaluate the correlations between serum sclerostin, bone sclerostin expression, and Wnt signaling. High-resolution immunofluorescence microscopy was performed across a subset of the population based on serum sclerostin levels. For these comparisons, representative examples were chosen from both pre-dialysis CKD (n = 6) and CKD 5D (n = 6) patients, as illustrated in the flowchart (Figure 2). From these two groups, subjects were further divided into those with either high serum sclerostin (58–125 pmol/L) or lower serum sclerostin, similar to healthy controls (18.6–36.0 pmol/L). As a control, sclerostin staining was also performed in healthy bone from non-pathologic fracture patients (20–30 pmol/L; n = 3).

First, CKD 5D patients with low serum sclerostin demonstrated low sclerostin expression in osteocytes, with expression levels that were similar to healthy controls (Figure 2A,B). Similarly, pre-dialysis CKD patients with low serum sclerostin also displayed low sclerostin bone expression (Figure 2C). In contrast, bone sclerostin expression was markedly increased in osteocytes from patients with high circulating sclerostin levels in both pre-dialysis CKD and CKD 5D, both in the trabecular and cortical regions (Figure 2D,F). Thus, circulating and bone sclerostin levels were correlated in both cohorts.

**Figure 3:**
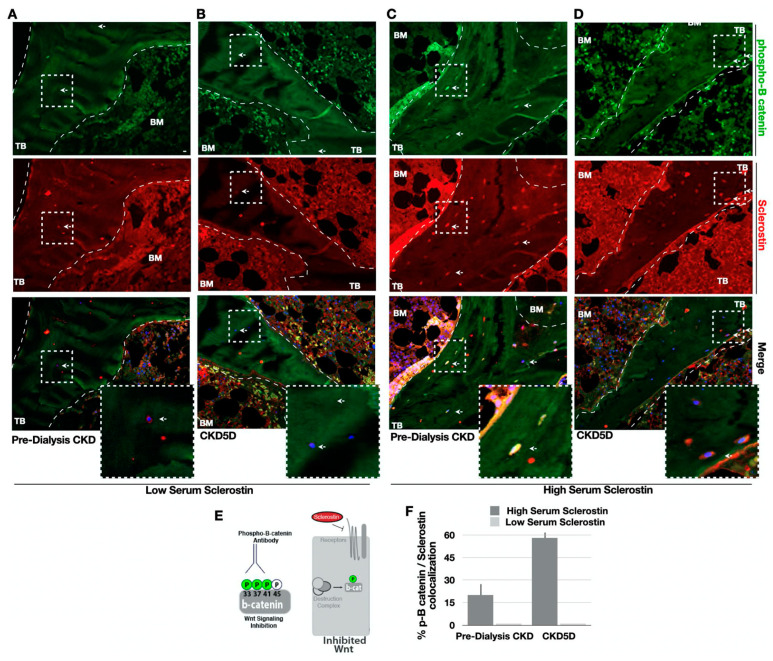
Phosphorylated-β-Catenin and Sclerostin Immunofluorescence Analyses in CKD Bone. (**A**–**D**) Bone stained with anti-sclerostin antibody (red), phosphorylated (phos) β-catenin (green), and DAPI (blue). Arrows denote corresponding cells with colocalized sclerostin and phos-β-catenin. Bottom panel indicates insets from dotted white boxes. (**E**) Model of the monoclonal antibody used here to recognize the phosphorylated (Ser33/37/Thr41) “inhibited” β-catenin peptide, which denotes an inhibition of the canonical Wnt pathway. (**F**) Quantification of immunofluorescence by the % of osteocytes positive for both phos-β-catenin and sclerostin relative to the total number of cells using bone unit area to normalize (n > 230 cells per condition). Biopsies were stained and processed side-by-side. Trabecular (TB) bone and bone marrow (BM) are indicated.

To determine whether sclerostin levels altered Wnt signaling activity in CKD bone, immunofluorescence colocalization analyses were performed with sclerostin and phos-β-catenin, which marks Wnt inhibition, and an unphosphorylated (active) β-catenin antibody, which marks Wnt activity. Low bone sclerostin expression correlated with low bone phos-β-catenin staining in both groups of CKD patients, as expected (Figure 3A,B). Patients with high sclerostin bone expression demonstrated significantly higher phos-β-catenin staining (Figure 3C,D). Furthermore, sclerostin-positive osteocytes were strongly colocalized with phos-β-catenin, which indicates Wnt signaling inhibition (Figure 3F).

**Figure 4:**
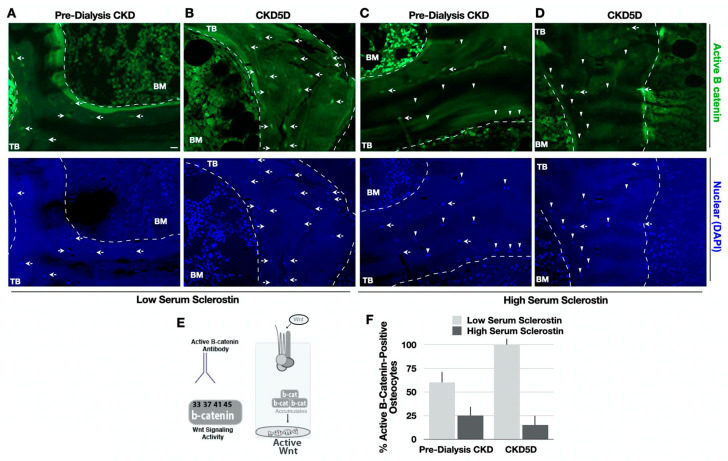
Active β-catenin Immunofluorescence Analyses in CKD bone. (**A**–**D**) Bone stained with active β-catenin (green) and DAPI (blue). Arrows denote corresponding cells with colocalized sclerostin and phos-β-catenin. (**E**) Model of the monoclonal antibody used to recognize the non-phosphorylated, stabilized β-catenin that is not tri-phosphorylated and functionally active in the canonical Wnt signaling pathway. (**F**) Quantification of immunofluorescence by the % of osteocytes positive for active β-catenin relative to the total number of cells using bone unit area to normalize (n > 230 cells per condition). Trabecular (TB) bone and bone marrow (BM) are indicated.

Subsequently, biopsies were stained with the unphosphorylated (active) β-catenin antibody (Figure 4). Pre-dialysis CKD patients with low circulating sclerostin levels had higher active β-catenin staining, indicating more Wnt signaling activity (Figure 4A). Similarly, CKD 5D patients with lower serum sclerostin also demonstrated higher Wnt signaling via active β-catenin staining (Figure 4B). In contrast, both CKD groups with high serum sclerostin levels showed significantly lower staining of active β-catenin (Figure 4D,F). 

## 4. Discussion

The present study represents the largest assessment of the impact of bone and circulating sclerostin on ROD across a pediatric CKD population. The prevalence of the subtypes of ROD across the cohort included adynamic bone (22%), osteitis fibrosa (16%), mixed uremic osteodystrophy (16%), osteomalacia (4.7%), normal bone turnover (39%), and mild ROD (2.3%). While the majority of the cohort had normal bone volume, mineralization defects were prevalent in pre-dialysis CKD and dialysis patients. Notably, the proportion of patients with normal bone turnover was higher in the earlier stages of CKD, while CKD 5D participants had higher bone turnover disease. Levels of serum calcium were in the normal range, hyperphosphatemia was present only in CKD 5D patients, and concentrations of PTH, alkaline phosphatase, and FGF-23 values were higher in the CKD 5D cohort, as previously described [30]. Sclerostin, an inhibitor of bone formation via Wnt signaling, was increased in circulation across the entire CKD cohort compared with controls, yet values were higher in dialysis patients. Serum sclerostin levels were inversely correlated with alkaline phosphatase in early CKD and with iPTH across the entire cohort. As expected, adynamic bone was associated with elevated serum sclerostin values, relative to normal or high bone turnover. Within the CKD 5D cohort, bone sclerostin levels were negatively associated with bone turnover and even correlated with a decreased risk of developing high bone turnover. Importantly, the elevated levels of sclerostin in circulation were mirrored in bone, and sclerostin-positive osteocytes colocalized with phos-β-catenin. Patients with low serum sclerostin had increased active β-catenin bone staining. In sum, these findings provide evidence that supports a model whereby sclerostin plays a role in the regulation of bone remodeling in pediatric CKD patients and may contribute to the pathogenesis of CKD-MBD.

Advances in technological approaches over the last decade have led to the revolutionary insight that osteocytes have multifaceted roles in bone physiology and organ crosstalk. In fact, the osteocyte is an endocrine organ, as they secrete factors that mediate signaling within their neighboring bone cell types and in distant tissues [35,36]. The dysfunctional activities of osteocytes have been linked to pathologies such as hypophosphatemic rickets, osteoporosis, and aging [11,37,38,39]. Osteocyte-secreted factors contribute to CKD-MBD progression, as demonstrated by genetically modified mouse models that elegantly dissect these molecular mechanisms [40,41,42,43,44]. Previously, the combination of bone biopsy and IHC analyses has identified that osteocyte-secreted factors are upregulated during the initial stages of CKD progression [45]. Similar findings have been reported across CKD stages in adults [25]. The present data supports the model whereby sclerostin, the predominant inhibitor of Wnt signaling in bone, is also misregulated in CKD-MBD in this pediatric population. Together, these findings provide mechanistic evidence that sclerostin regulates bone remodeling and represents a key element in early CKD stages and disease progression. 

Serum sclerostin has been measured in pediatric CKD [46,47], and Guven et al. report negative associations with alkaline phosphate levels, consistent with the current findings. However, the absence of bone biopsies in previous studies made it difficult to conclusively evaluate bone sclerostin levels in these patients. The present analyses of bone histomorphometry with serum and bone sclerostin provide several new insights into the roles of sclerostin on bone turnover in pediatric CKD-MBD. First, elevated serum and bone sclerostin levels were inversely correlated with low bone turnover. Second, circulating sclerostin corresponded with bone sclerostin, which colocalized with phos-β-catenin in osteocytes. Third, serum sclerostin was inversely correlated with osteocytic staining of an unphosphorylated β-catenin antibody. These analyses provide additional evidence to support a model whereby skeletal sclerostin expression regulates bone tissue remodeling through the Wnt pathway. Future investigations will be required in order to conclusively discriminate between paracrine and autocrine-derived signaling axes [18].

Elevated sclerostin levels occur prior to the rise of PTH or the decline of kidney function, as previously reported [12]. In line with this model, it is interesting to note the previous work showing that in parathyroidectomized patients, sclerostin levels normalized faster than markers of bone turnover [7,48]. Our current findings suggest that circulating sclerostin levels may be a useful biomarker and could help to improve the global understanding of the effects of PTH on skeletal tissues. Indeed, the ratio of sclerostin to PTH provided further insights into the skeletal resistance to PTH in these patients. The correlation of sclerostin with parameters of mineralization and turnover was stronger when we applied sclerostin to the PTH ratio. In the pre-dialysis CKD cohort, we report that the increased ratio of sclerostin to PTH was associated with a decreased risk of developing a mineralization defect. In dialysis patients, ratios of sclerostin to PTH correlated with a decreased chance of high bone turnover. Since the skeletal resistance to PTH differs according to the specific subtype of ROD in dialysis patients [49], the ratio of sclerostin to PTH may be utilized as an additional variable to further define such a response.

Our findings are in line with others showing that sclerostin levels remain higher than in control subjects, despite the rise in PTH during CKD progression [50,51]. Kidney transplant patients demonstrate a remarkably rapid decrease in serum sclerostin, occurring even faster than changes in PTH [24,52,53]. Even following transplantation, sclerostin eventually begins to rise in the case of hyperparathyroidism, along with FGF-23, uremic toxins, and various inflammatory cytokines [48,54,55,56,57]. While the links between PTH and sclerostin remain to be completely understood by longitudinal studies, it is clear that there is a distinct relationship where overproduction of PTH is generally correlated with lower serum sclerostin. Further explorations of the dynamic interplay are warranted.

In adult CKD, sclerostin was a good predictor of low bone turnover and bone volume, while PTH and FGF-23 predicted high bone turnover [25,58]. Recent work highlights that a progressive increase in FGF-23 and sclerostin in blood and bone is associated with a decrease in kidney function, which suggests using the correlations of bone turnover and sclerostin or FGF-23 could improve patient management in CKD patients with turnover abnormalities [59,60,61,62,63]. In our pediatric population, adynamic bone was associated with elevated serum sclerostin compared with normal or high bone turnover. Importantly, bone sclerostin correlated with a decreased risk of developing high bone turnover disease in the CKD 5D cohort. Patients with pre-dialysis CKD showed significant inverse associations between indices of bone turnover and serum sclerostin. Interestingly, it was only in CKD 5D that bone sclerostin significantly correlated with bone turnover, which suggests that the role of residual renal function in pre-dialysis CKD patients remains to be defined. Altogether, these findings support the emerging evidence that secreted factors from osteocytes are among the earliest signals that contribute to the beginning stages of CKD-MBD progression.

ROD is a complex systemic disorder of bone structure, metabolism, and cellular function. Treatments for ROD have been limited by the intrinsic complexity of the disease and a lack of molecular mechanistic knowledge of organ crosstalk at the cellular level. Our findings show the value of bone biopsy as an approach to understanding how the cellular signaling emanating from osteocytes contributes to bone disease in CKD. FDA-approved inhibitors of osteocytic proteins such as FGF-23 and sclerostin have been used to treat bone diseases [37]. Given this, future studies of larger patient populations are among the top priorities for generating new treatments and biomarkers for CKD-MBD in children and adults.

## Figures and Tables

**Table 1:** Clinical Characteristics of Pre-Dialysis CKD and CKD 5D Patients.

	Pre-Dialysis CKD	CKD 5D	*p* Value
N (%)	24	29	
Age, (median (IQR)	15.0 (12.7, 17.1)	18.3 (16.4, 19.7)	<0.0001
Gender, (n (%))			0.8
Male	23 (68)	37 (70)	
Female	11 (32)	16 (30)	
Race, (n (%))			0.01
Black	2 (6)	5 (9)	
White	14 (41)	6 (11)	
Hispanic	18 (53)	39 (74)	
Asian		3 (6)	
Disease, (n (%))			0.0003
CAKUT	23 (68)	15 (28)	
GN	10 (29)	21 (40)	
Unknown	1 (3)	17 (32)	
CKD Stage, (n (%))			
Stage 2 & 3	28 (82)		
Stage 4	5 (14)		
Stage 5	1 (3)		
Dialysis, (n (%))			
HD		25 (45)	
PD		29 (55)	

IQR: interquartile range. CKD: Chronic kidney disease. HD: hemodialysis. PD: peritoneal dialysis.

**Table 2:** Biochemical Determinations of Pre-Dialysis and CKD 5D Patients.

	Pre-Dialysis CKD	CKD 5D	*p* Value
Ca (mg/dL)	9.4 (9.0, 9.7)	9.2 (8.7, 9.7)	0.2
P0_4_ (mg/dL)	4.8 (4.0, 5.3)	6.3 (5.2, 7.6)	<0.0001
P0_4_ Z-score	1.5 (0.3, 2.3)	5.0 (2.9, 7.7)	<0.0001
Alk Phos (IU/L)	171 (110, 265)	150 (92, 283)	0.5
25-(OH) Vitamin D (ng/mL)	26 (24, 30)	21 (13, 30)	0.1
iPTH (pg/mL)	95 (50, 159)	411 (211, 991)	<0.0001
Intact FGF-23 (pg/mL)	95 (64, 140)	1195 (286, 5718)	<0.0001
C-terminal FGF-23 (RU/mL)	199 (101, 344)	1463 (705, 5577)	<0.0001
Scl (pmol/L)	40.3 (34.2, 52.6)	66.9 (48.3, 87.2)	<0.0001

Values are expressed as mean and interquartile range (IQR). Scl: sclerostin.

**Table 3:** Bone Histomorphometry by TMV Classification in Pre-Dialysis CKD and CKD 5D Patients.

	Pre-Dialysis CKD	CKD 5D	*p* Value	Reference Range
Turnover				
Bone formation rate (BFR/BS; μm^3^/μm^2^/year)	19.4 (12.2, 44.8)	30.9 (5.1, 61.8)	0.8	8.0–73.4
Eroded Surface (ES/BS; %)	4.4 (2.5, 7)	9.8 (5.8, 13.5)	0.0001	0.5–4.3
Mineralization				
Osteoid Volume (OV/BV; %)	4.9 (1.6, 7.8)	4.4 (1.7, 7.6)	0.6	0.2–5.8
Osteoid thickness (O.Th; μm)	10.9 (7.7, 15.4)	9.7 (6.9, 11.8)	0.1	2.0–13.2
Osteoid surface (OS/BS; %)	27.2 (12.5, 39.9)	30.4 (16.7, 43.7)	0.5	4.3–37.0
Osteoid maturation time (OMT; day)	13.2 (9.3, 18.5)	11.8 (8.5, 15)	0.2	1.2–11.5
Mineralization lag time (MLT; day)	34.3 (18.5, 52.4)	28.4 (19.5, 75.4)	0.9	2.3–63.8
Volume				
Bone volume (BV/TV; %)	27.2 (22.7, 32.3)	31.5 (26.2, 36.4)	0.04	8.9–34.4
Trabecular number (Tb.N; mm)	2.2 (1.8, 2.4)	2.2 (1.9, 2.4)	0.3	1.1–2.2
Trabecular Separation (Tb.Sp; μm)	334 (307, 422)	325 (271, 361)	0.1	351–737
Osteoblast Surface (ObS/BS; %)	2.7 (1.2, 17.3)	5.5 (2, 9.9)	0.5	
Osteoclast Surface (OcS/BS; %)	0.8 (0.3, 1.1)	1.9 (1.2, 3.4)	0.0001	

**Table 4:** Sclerostin PTH Ratio Analyses.

	Pre-Dialysis CKD			CKD 5D		
	PTH	Alk Phos	Bone FGF-23	PTH	Alk Phos	Bone FGF-23
Serum Scl	−0.42 *p* = 0.02	0.11 *p* = 0.5	−0.36 *p* = 0.1	−0.31 *p* = 0.04	−0.37 *p* = 0.01	0.12 *p* = 0.4
Bone Scl	0.28 *p* = 0.3	−0.29 *p* = 0.3	0.18 *p* = 0.5	−0.29 *p* = 0.06	−0.34 *p* = 0.02	0.5 *p* = 0.0002
Scl/PTH ratio	−0.93 *p* < 0.0001	−0.23 *p* = 0.2	0.19 *p* = 0.4	−0.93 *p* < 0.0001	−0.44 *p* = 0.004	0.12 *p* = 0.4

## Data Availability

All data will be available upon email request.

## Supplementary Materials

The following supporting information can be downloaded at: https://www.mdpi.com/article/10.3390/nu15194127/s1, Table S1. Spearman Correlation between Biochemical Parameters, Bone Variables and Skeletal Sclerostin.
