# Supplementary material for: Sclerostin, Osteocytes, and Wnt Signaling in Pediatric Renal Osteodystrophy
Source: Nutrients. 2023 Sep 25;15(19):4127. doi: 10.3390/nu15194127 (PMC10574198; doi:10.3390/nu15194127)
Supplement: Supplementary file 1 [file nutrients-15-04127-s001.zip › nutrients-2570335-supplementary.pdf]

Supplementary Material

Supplementary Table S1. Spearman Correlation between Biochemical Parameters, Bone Variables and Skeletal Sclerostin

	Pre-Dialysis CKD								
	**BFR/BS**	**ES/BS**	**OV/BV**	**OTH**	**OS/BS**	**OMT**	**MLT**	**MS/BS**	**MS/OS**
**Serum Scl**	-0.31	**-0.44**	-0.31	-0.31	-0.25	-0.28	-0.15	**-0.40**	0.19
	p=0.07	**p=0.01**	p=0.07	p=0.07	p=0.2	p=0.1	p=0.4	**p=0.02**	p=0.4
**Bone Scl**	0.34	-0.15	-0.03	-0.08	0.09	0.12	0	0.45	0.12
	p=0.2	p=0.5	p=0.9	p=0.8	p=0.7	p=0.6	p=1	p=0.05	p=0.6
**PTH**	**0.36**	**0.40**	**0.65**	**0.64**	**0.66**	**0.43**	**0.38**	**0.40**	-0.12
	**p=0.04**	**p=0.03**	**p<0.0001**	**p<0.0001**	**p<0.0001**	**p=0.01**	**p=0.03**	**p=0.02**	p=0.6
**Scl/ PTH**	**-0.42**	**-0.5**	**-0.6**	**-0.59**	**-0.6**	**-0.42**	**-0.33**	**-0.49**	0.16
**ratio**	**p=0.02**	**p=0.003**	**p=0.0003**	**p=0.0004**	**p=0.0003**	**p=0.02**	**p=0.06**	**p=0.004**	p=0.5
**Intact**	**0.5**	0.11	0.21	0.24	0.22	-0.003	-0.2	**0.5**	**0.58**
**FGF-23**	**p=0.004**	p=0.5	p=0.2	p=0.2	p=0.2	p=1	p=0.3	**p=0.004**	**p=0.03**
**C-term**	0.34	0.19	0.14	0.08	0.18	-0.07	-0.1	**0.3**	0.19
**FGF-23**	p=0.1	p=0.3	p=0.4	p=0.6	p=0.3	p=0.7	p=0.6	**p=0.08**	p=0.4
**Bone**	0.42	0.04	0.002	-0.28	-0.002	-0.25	-0.25	0.23	0.07
**FGF-23**	p=0.1	p=0.9	p=1.0	p=0.2	p=1	p=0.3	p=0.3	p=0.3	p=0.8

	CKD 5D								
	**BFR/BS**	**ES/BS**	**OV/BV**	**OTH**	**OS/BS**	**OMT**	**MLT**	**MS/BS**	**MS/OS**
**Serum Scl**	-0.15	-0.08	**-0.38**	**-0.35**	**-0.42**	-0.16	-0.13	-0.19	0.12
	p=0.3	p=0.6	**p=0.005**	**p=0.01**	**p=0.002**	p=0.3	p=0.4	p=0.2	p=0.4
**Bone Scl**	**-0.34**	-0.23	-0.24	**-0.3**	-0.18	0.002	0.20	**-0.33**	-0.21
	**p=0.02**	p=0.1	p=0.1	**p=0.03**	p=0.2	p=1	p=0.2	**p=0.03**	p=0.1
**PTH**	**0.50**	**0.39**	**0.37**	**0.51**	**0.36**	**-0.07**	-0.27	**0.46**	0.23
	**p=0.001**	**p=0.009**	**p=0.01**	**p=0.0003**	**p=0.02**	**p=0.7**	p=0.1	**p=0.002**	p=0.1
**Scl/ PTH**	**-0.47**	**-0.35**	**-0.42**	**-0.53**	**-0.43**	0.04	0.17	**-0.44**	-0.14
**ratio**	**p=0.002**	**p=0.02**	**p=0.004**	**p=0.0002**	**p=0.003**	p=0.8	p=0.3	**p=0.004**	p=0.4
**Intact**	-0.06	-0.05	**-0.44**	-0.27	**-0.51**	**-0.54**	**-0.4**	-0.16	0.23
**FGF-23**	p=0.7	p=0.7	**p=0.002**	p=0.07	**p=0.0004**	**p=0.0003**	**p=0.01**	p=0.3	p=0.1
**C-terminal**	p=-0.17	-0.05	**-0.32**	-0.24	**-0.35**	**-0.41**	-0.23	-0.26	0.15
**FGF-23**	p=0.2	p=0.7	**p=0.02**	p=0.09	**p=0.01**	**p=0.01**	p=0.1	p=0.08	p=0.3
**Bone**	**-0.30**	-0.14	**-0.44**	**-0.51**	**-0.40**	**-0.44**	-0.21	**-0.30**	0.05
**FGF-23**	**p=0.04**	p=0.3	**p=0.001**	**p=0.0001**	**p=0.003**	**p=0.003**	p=0.2	**p=0.04**	p=0.7

P < 0.05 indicates significance
